# Impact of invasive infections on clinical outcomes in acute pancreatitis: early predictive factors and implications for prophylactic anti-infective therapy

**DOI:** 10.1186/s13099-024-00671-3

**Published:** 2025-01-19

**Authors:** Fabienne Bender, Theresa König, Matthias Hecker, Moritz Fritzenwanker, Jacqueline Braun, Joern Pons-Kühnemann, Matthias Wolff, Andreas Hecker, Martin Reichert

**Affiliations:** 1 Department of General, Visceral, Thoracic and Transplant Surgery, University Hospital of Giessen, Rudolf-Buchheim-Strasse 7, 35392 Giessen, Germany; 2 Department of Pulmonary and Critical Care Medicine, University Hospital of Giessen, Klinikstrasse 33, 35392 Giessen, Germany; 3 Institute of Medical Microbiology, Justus-Liebig-University of Giessen, Schubertstrasse 81, 35392 Giessen, Germany; 4 Medical Statistics, Institute of Medical Informatics, Justus-Liebig-University of Giessen, Rudolf-Buchheim-Strasse 6, 35392 Giessen, Germany; 5 Department of Anaesthesiology, Intensive Care Medicine and Pain Therapy, University Hospital of Giessen, Rudolf-Buchheim-Strasse 7, 35392 Giessen, Germany

**Keywords:** Acute pancreatitis, Pathogens, Antibiotic prophylaxis, Ascites, White blood cell count

## Abstract

**Background:**

The use of antibiotic therapy in acute pancreatitis remains controversial and is currently recommended only for confirmed infections of peripancreatic necrosis. However, reliable early predictors of septic complications and unfavorable outcomes are substantially lacking.

**Methods:**

Patients with acute pancreatitis were retrospectively reviewed and divided into two groups: one with a septic course defined by pathogen detection [GERM(+)] and one without [GERM(-)]. After propensity score matching, both groups were compared regarding clinical outcomes. Early predictors of pathogen detection were evaluated by multivariate analysis.

**Results:**

424 patients with acute pancreatitis were included. After propensity score matching 123 GERM(-) patients were compared to 74 GERM(+) patients. GERM(+) patients demonstrated significantly worse clinical outcomes with higher rate of intensive care treatment (59.5% vs. 35.0%; *p *= 0.0011) and consecutive longer stay in intensive care unit (11.5 ± 25.2d vs. 3.0 ± 7.9d; *p *= 0.0007), longer in-hospital stay (26.8 ± 22.0d vs. 14.7 ± 15.0d; *p* = 0.0003) as well as worse results in the composite outcome length of in-hospital stay > 15d or death (67.6% vs. 31.7%; *p* < 0.0001). Prescence of ascites and elevated white blood cell count at the onset of acute pancreatitis were identified as significant predictive factors in the early disease associated with invasive infection and pathogen detection. The most frequently detected pathogens were commensals of the gastrointestinal tract, observed in 70.7% of the examined body fluids and 50.7% of the examined blood cultures.

**Conclusions:**

Detection of pathogens is associated with unfavorable clinical outcomes in acute pancreatitis. The presence of ascites and elevated white blood cell count at onset of acute pancreatitis are significant predictive factors indicating the risk of invasive infection with relevant bacterial load. Thus, an aggressive, early anti-infective strategy against pathogens of intestinal origin should be considered in these cases and may improve patient outcomes.

**Supplementary Information:**

The online version contains supplementary material available at 10.1186/s13099-024-00671-3.

## Introduction

Acute pancreatitis (aP) is a common disease in industrialized countries, characterized by high rates of mortality, especially in severe cases [1]. The revised 2012 Atlanta classification defines three degrees of severity: mild, moderate, and severe. These encompass local or systemic complications with organ failure [2]. Despite advances in local therapy strategies, such as the *step-up approach* [3] and modern intensive care therapy, mortality rates are still approaching 35% in patients with severe aP [4].

During the natural course of the disease, two peaks of mortality arise [2]. The first typically occurs within the first two weeks, driven by severe sterile inflammation as part of the *systemic inflammatory response syndrome* (SIRS). This is triggered by cytokine cascade activation, resulting in early (multi-) organ failure. Thereafter, the *compensatory anti-inflammatory response syndrome* (CARS) underlies the second peak of mortality [5]. In this context, translocation of intestinal bacteria leads to infection of (peri-) pancreatic necroses [6, 7] and other infectious complications in distant organs such as pneumonia [7–9].

However, distinguishing between severe non-infection-triggered SIRS and infectious complications remains challenging in clinical practice [10–12]. Diagnosis often relies on indirect signs of infected necrosis, such as characteristic imaging findings [10, 13] or clinical deterioration of the patients [14]. Nevertheless, in patients with confirmed infected pancreatic necrosis [10, 15] antibiotic therapy is uncontroversial [10, 12, 13, 16, 17]. In other cases, the use of antibiotics remains debated [7, 13, 18]. This restrictive approach for prophylactic antibiotics in clinical routine [10, 12, 17–19] is driven by the risk of significant adverse effects, including bacterial or fungal overgrowth [9] and the development of multi-drug resistant pathogens [20], which negatively impact on morbidity and mortality of the patients [21–23]. This underscores the urgent need to identify and implement risk factors in clinical practice that guide the appropriate and targeted use of antibiotics at an early stage of the disease [13, 18]. This approach aims to prevent infectious complications and reduce morbidity and mortality associated with aP.

The aim of this study was to assess the impact of pathogens on clinical outcomes in patients with aP and to identify early risk factors predictive of bacterial load and infection. These factors determine the necessity of initiating targeted prophylactic antibiotic therapy at an early stage of the disease, aiming to improve patient outcomes by preventing the progression of infection.

## Methods

This retrospective, exploratory single-center study was conducted in line with the latest Declaration of Helsinki and was approved by the local ethics committee of the University of Giessen medical faculty (approval No. 174/20). Data collection, manuscript drafting, and submission adhered to the COPE (Committee on Publication Ethics) guidelines as well as the Strengthening the reporting of observational studies in epidemiology (STROBE) statement [24]. All patients received treatment according to the institutional standard of care.

Patients (≥12 years of age) who underwent in-hospital treatment from 01/2010 to 12/2021 at the University Hospital of Giessen for aP diagnosed according to the revised Atlanta classification were included into the study [2]. Patients with recurrent aP were included multiple times as single cases if there were no signs of a chronic disease and if there was significant temporal distance between hospital discharge and readmission, provided there were no signs of persistent pancreatitis upon readmission. The morphological differentiation of aP was conducted according to the revised Atlanta Classification, distinguishing between edematous and necrotizing aP [2]. Even for patients with mild clinical symptoms who did not undergo further imaging or specific treatment, aP was retrospectively classified as edematous pancreatitis. According to the revised Atlanta Classification, the severity of aP was categorized into the three groups: mild, moderate, and severe [2].

Patient data were retrospectively collected from the prospectively maintained institutional databases. These included general patient characteristics, etiology and severity of aP, treatment information, patient outcomes with length of hospital stay, in-hospital morbidity and mortality. Detection of pathogens in blood culture, bile, (peri-) pancreatic necrosis, ascites and/or pleural effusion indicated invasive infection. Laboratory markers were assessed at onset of aP as well as on treatment days 1 and 3. White blood cell count (WBC) and C-reactive protein (CRP) were used as routine markers for systemic inflammation and infection; serum lipase, amylase and total bilirubine at onset indicated pancreatic injury and etiology of aP; pathological serum creatinine, Quick’s value and total bilirubine indicated renal and liver dysfunction during aP treatment. The composite outcome, which includes in-hospital stay longer than 15 days (aligned with the mean length of stay for the entire cohort) or mortality, served as the surrogate parameter for clinical outcome in patients with aP. Retrospective availability of data was > 97%.

Microorganisms detected during the hospital stay were taken into consideration and were revised regarding their resistance profile according to current EUCAST (The European Committee on Antimicrobial Susceptibility Testing) guidelines (breakpoint tables for interpretation of minimum inhibitory concentrations and zone diameters, version 14.0, 2024, EUCAST antifungal clinical breakpoint table, v 10.0, 2020, and expected resistant and susceptible phenotypes, v 1.2, 2023; http://www.eucast.org) as previously described [25].

### Institutional treatment standards of acute pancreatitis

As recommended in current guidelines, the management of aP therapy included fluid resuscitation, analgesia and symptomatic therapy [4, 26, 27]. Patients received enteral nutrition whenever feasible. Those with severe aP, critical illness, or signs of organ dysfunction were generally treated in the intensive care unit (ICU).

Anti-infective therapy was used restrictively. Empirical antibiotic therapy was initiated in patients showing signs of infection and subsequently adjusted based on microbiological findings. In cases of biliary etiology, bile duct clearance was performed as promptly as possible utilizing endoscopic retrograde cholangiopancreatography (ERCP). Cross-sectional imaging was indicated in cases of severe aP, including those with organ failure, suspected infection or local complications, such as infected necrosis. The latter was treated following the *step-up approach* [3]. Surgical intervention was indicated as second-line treatment when previous endoscopic or radiological interventions had failed or if severe surgical complications arose.

### Statistical analyses

The patient cohort was divided into two groups regarding the diagnosis of invasive infection by detection of pathological microorganisms in body fluids during the hospital treatment of aP into a germ-negative [GERM(-); *n* = 332] and germ-positive [GERM(+); *n* = 92] group of patients.

Statistical analyses were performed using *GraphPad Prism* (Version 10 for Windows, GraphPad Software, San Diego, CA, USA; www.graphpad.com). Fisher’s exact or Pearson’s $\:\chi\:$^2^ test were performed for intergroup comparisons of categorical data, Student’s t-test was used for two-group comparisons of continuous variables. One-way ANOVA was used to evaluate global effects in multiple-group comparisons. If applicable, differences between all groups were investigated post-hoc with Tukey’s test after correction for multiple testing. Data in tables are given in n (%) or means ± standard deviations. Column bar graphs in figures indicate means and standard deviations.

Because of imbalances in basic patient characteristics between both groups, 1:2 propensity score pair matching (PSM) with a match tolerance = 0.1 was performed using the package *Match it* with *R* (version 4.3.2). The propensity score was calculated with relevant parameters of patient characteristics and relevant characteristics of aP (including etiology and severity of aP, cardio-vascular disease, chronic kidney disease and Charlson comorbidity index). Group comparisons of the propensity score matched cohorts [^psm^GERM(-) versus ^psm^GERM(+)] were performed as described above.

Simple linear regression was used for univariable analysis and multiple linear regression for multivariable analysis to evaluate relevant early predictors and risk factors associated with bacterial load and worse clinical outcome of patients with aP. Therefore, variables with Spearman’s r^2^ > 0.05 and *p* < 0.01 in univariable analysis were considered as relevant for inclusion into multivariable analysis. Statistical significance was indicated by *p*-value ≤ 0.05.

## Results

### Patient cohort and baseline characteristics

424 patients underwent in-hospital treatment of aP during the observational period and were included in the study. After subdividing the total cohort, 92 patients were found to have an invasive infection, as evidenced by positive bacterial cultures from blood, bile fluid, pleural effusion, ascites, and/or pancreatic necrosis [GERM(+)], whereas 332 patients did not [GERM(-)]. Baseline characteristics of both patient cohorts were significantly different regarding comorbidities (including higher rates of chronic diseases and higher scores in Charlson comorbidity index: 3.1 ± 2.7 versus 4.2 ± 2.7; *p* = 0.0008), etiology and severity (severe: 3.0% versus 19.6%; *p* < 0.0001) of aP, to the disadvantage of the GERM(+) group of patients (Table 1).

**Table 1:** Characteristics of patients, characteristics of acute pancreatitis and clinical outcome before and after propensity score matching

Variable	All patients			Propensity score-matched patients		
	Germ(–) *n*=332	Germ(+) *n*=92	*p* value	^psm^Germ(–) *n*=123	^psm^Germ(+) *n*=74	*p* value
**Patient characteristics**						
Male gender [n]	183 (55.1%)	52 (56.5%)	0.9057	82 (66.7%)	40 (54.1%)	0.0957
Age [years]	57.0 ± 17.9	60.8 ±17.5	0.0763	63.3 ± 16.1	59.9 ± 18.0	0.1675
BMI [kg/m^2^]	28.2 ± 6.4	27.2 ± 6.7	0.1916	27.2 ± 5.5	27.5 ± 7.2	0.7547
Active abuse [n]						
Alcohol Smoking	82 (24.7%) 90 (27.1%)	21 (22.8%) 13 (14.1%)	0.7843 0.0092	31 (25.2%) 36 (29.3%)	12 (16.2%) 13 (17.6%)	0.1572 0.0883
Liver cirrhosis [n]	4 (1.2%)	2 (2.2%)	0.6148	1 (0.8%)	2 (2.7%)	0.5576
Diabetes mellitus [n]	61 (18.4%)	18 (19.6%)	0.7645	32 (26.0%)	15 (20.3%)	0.3926
Cardiovasculary disease [n]	193 (58.1%)	67 (72.8%)	0.0111	87 (70.7%)	50 (67.6%)	0.6360
Chronic lung disease [n]	51 (15.4%)	20 (21.7%)	0.1569	27 (22.0%)	17 (23.0%)	0.8618
Chronic kidney disease [n]	27 (8.1%)	20 (21.7%)	0.0006	20 (16.3%)	13 (17.6%)	0.8452
Cerebrovasculary disease [n]	14 (4.2%)	6 (6.5%)	0.4030	7 (5.7%)	4 (5.4%)	1.000
Charlson comorbidity index [score]	3.1 ± 2.7	4.2 ± 2.7	0.0008	4.5 ± 2.9	4.0 ± 2.6	0.2680
**Characteristics of acute pancreatitis**						
Etiology [n]			0.0021			0.2308
Biliary Post-ERCP Ethyltoxic Postoperative Idiopathic Other	168 (50.6%) 69 (20.8%) 15 (4.5%) 26 (7.8%) 27 (8.1%) 27 (8.1%)	29 (31.5%) 20 (21.7%) 5 (5.4%) 16 (17.4%) 6 (6.5%) 16 (17.4%)		46 (37.4%) 36 (29.3%) 10 (8.1%) 14 (11.4%) 10 (8.1%) 7 (5.7%)	27 (36.5%) 18 (24.3%) 5 (6.8%) 12 (16.2%) 2 (2.7%) 10 (13.5%)	
Morphological classification [n]			<0.0001			0.2681
Edematous Necrotizing	305 (91.9%) 27 (8.1%)	62 (67.4%) 30 (32.6%)		102 (82.9%) 21 (17.1%)	56 (75.7%) 18 (24.3%)	
Severity * [n]			<0.0001			0.3373
Mild Moderate Severe	280 (84.3%) 42 (12.7%) 10 (3.0%)	44 (47.8%) 30 (32.6%) 18 (19.6%)		83 (67.5%) 31 (25.2%) 9 (7.3%)	43 (58.1%) 22 (29.7%) 9 (12.2%)	
**Outcomes and length of hospitalization**						
Antibiotic therapy [n]	139 (41.9%)	39 (42.4%)	1	66 (53.7%)	31 (41.9%)	0.1410
Interventional (endoscopic or radiologic) treatment of (peri-) pancreatic necroses [n]	23 (6.9%)	25 (27.2%)	<0.0001	13 (10.6%)	17 (23.0%)	0.0243
Surgical intervention of (peri-) pancreatic necroses [n]	3 (0.9%)	19 (20.7%)	0.0003	3 (2.4%)	13 (17.6%)	<0.0001
Pleural effusion [n]	84 (25.3%)	63 (68.5%)	<0.0001	47 (38.2%)	46 (62.2%)	0.0012
Ascitic fluid [n]	78 (23.5%)	59 (64.1%)	<0.0001	38 (30.9%)	43 (58.1%)	0.0003
Intensive care treatment [n]	74 (22.3%)	62 (67.4%)	<0.0001	43 (35.0%)	44 (59.5%)	0.0011
Length of stay on ICU [d]	1.5 ± 5.1	13.7 ± 24.7	<0.0001	3.0 ± 7.9	11.5 ± 25.2	0.0007
Length of In-hospital stay ^§^ [d]	11.3 ± 10.7	29.8 ± 24.9	<0.0001	14.7 ± 15.0	26.8 ± 22.0	0.0003
Composite outcome ^#^ [n]	65 (19.6%)	66 (71.7%)	<0.0001	39 (31.7%)	50 (67.6%)	<0.0001
In-hospital mortality [n]	8 (2.4%)	15 (16.3%)	<0.0001	7 (5.7%)	7 (9.5%)	0.3930

Data are given in mean and SD or n (%). PSM = propensity score matching; BMI = Body mass index; ERCP = Endoscopic retrograde cholangiopancreatography; ICU = Intensive care unit

* Severity of acute pancreatitis was assessed concerning the revised Atlanta classification of 2012 [2]

§ Excluding patients who suffered from in-hospital mortality

# The composite outcome was defined as a prolonged in-hospital stay > 15 days (based on the mean length of stay of the entire cohort) or mortality and was used as the surrogate indicator for overall acute pancreatitis outcome

### Outcome analysis of the unmatched patient cohort

Patients from the GERM(+) group had significantly higher rates of pleural effusion and ascites, as well as poorer outcome in terms of in-hospital mortality rate. Additionally, the rate of intensive care treatment was higher in the GERM(+) cohort, as were the lengths of stay in the ICU and total hospital stay. This resulted in a higher incidence of worse composite outcome, including prolonged hospitalization beyond the mean duration of hospitalization of the total patient cohort or in-hospital mortality (19.6 vs. 71.7%; *p* < 0.0001; Table 1). These findings suggest poorer clinical outcomes in patients with invasive infection, evidenced by positive bacterial cultures in body fluids. Supplement 1 provides evidence of higher serological markers of inflammation and infection, WBC and CRP, in GERM(+) patients. Although systemic lipase and amylase values at onset and during the early treatment phase did not differ between the groups (Supplement 2), creatinine values and serum total bilirubin were markedly increased, Quick values indicating prothrombin time as a global parameter for liver (dys-) function were significantly decreased in patients from the GERM(+) group (Supplement 3). This indicates the systemic impact of aP, organ failure, and the critical illness of these patients.

### Patient characteristics and outcome analysis after propensity score matching

**Fig. 1:**
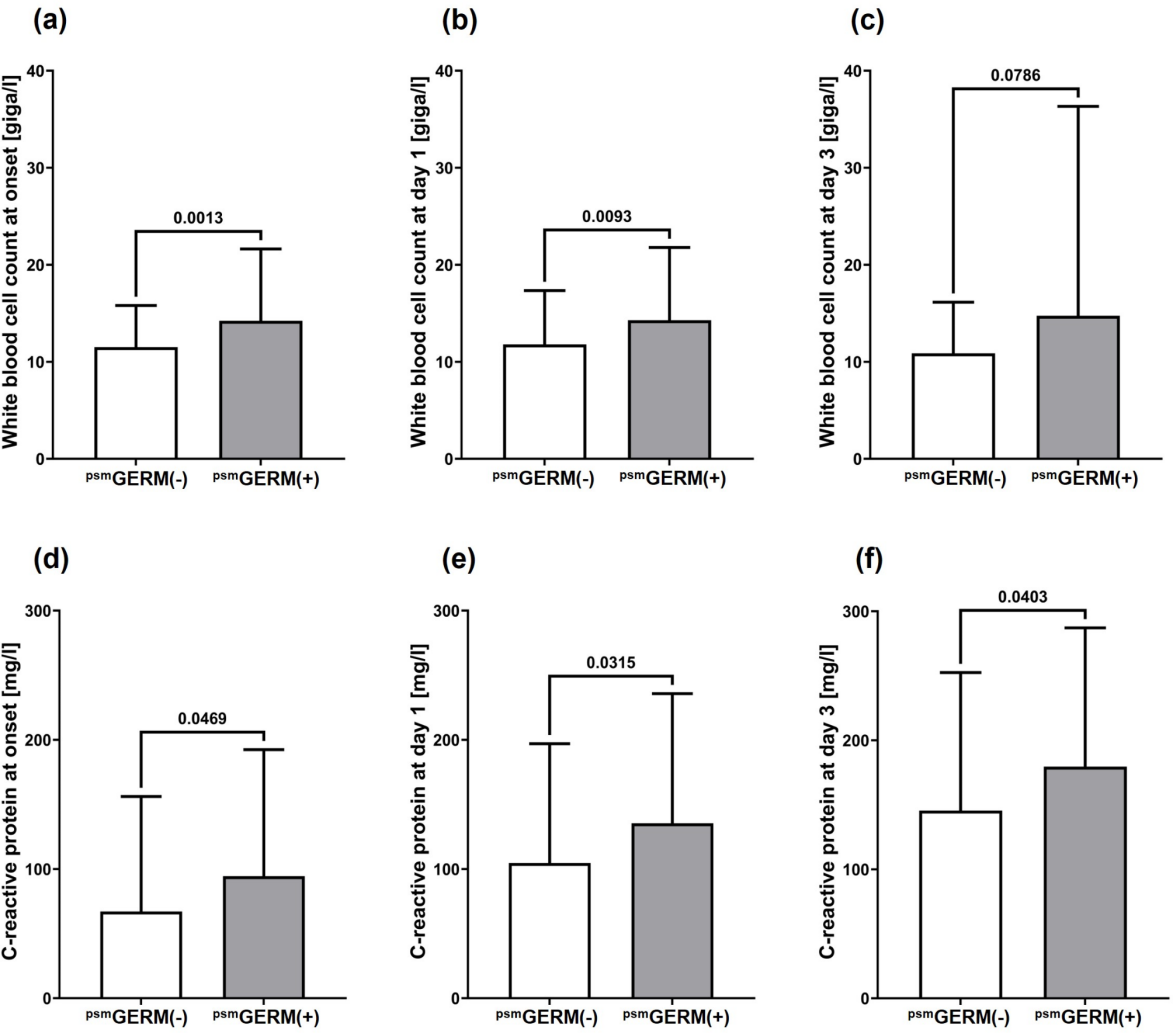
Serological markers for systemic inflammation or infection. Columns indicate means and bars represent the respective standard deviations of white blood cell counts **(a-c)** and C-reactive protein values **(d-f)** in peripheral blood of the propensity score matched (psm) patient cohorts without [^psm^GERM(-)] and with [^psm^GERM(+)] pathogen detection during acute pancreatitis therapy at onset of acute pancreatitis **(a**,** d)** and at in-hospital treatment day 1 **(b**,** e)** and day 3 **(c**,** f)**. The corresponding p values for each two-group comparison are indicated in the respective figures

**Fig. 2:**
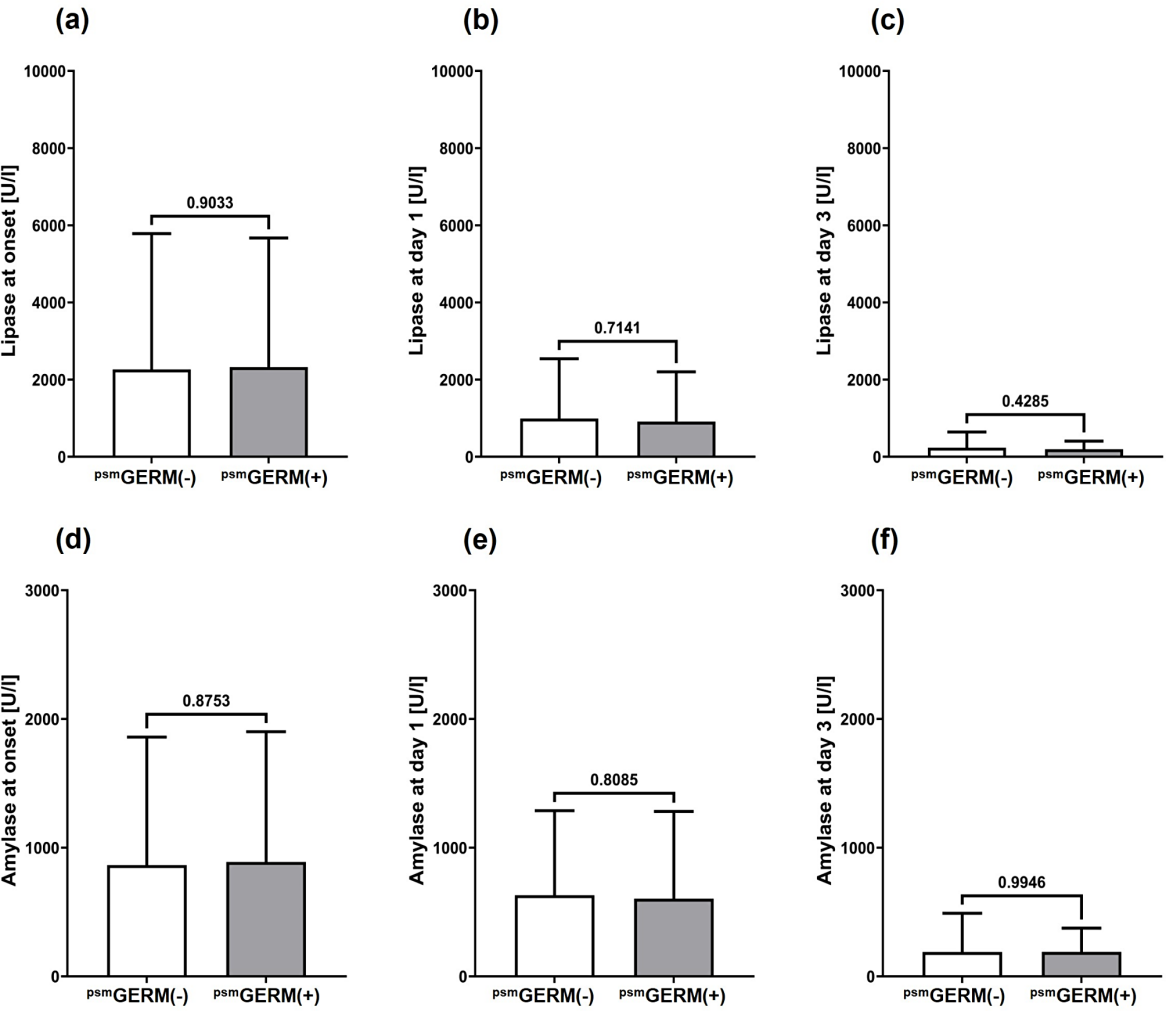
Serum lipase and amylase as serological markers for acute pancreatitis. Columns indicate means and bars represent the respective standard deviations of serum lipase **(a-c)** and serum amylase values **(d-f)** in peripheral blood of the propensity score matched (psm) patient cohorts without [^psm^GERM(-)] and with [^psm^GERM(+)] pathogen detection during acute pancreatitis therapy at onset of acute pancreatitis **(a**,** d)** and at in-hospital treatment day 1 **(b**,** e)** and day 3 **(c**,** f)**. The corresponding p values for each two-group comparison are indicated in the respective figures

**Fig. 3:**
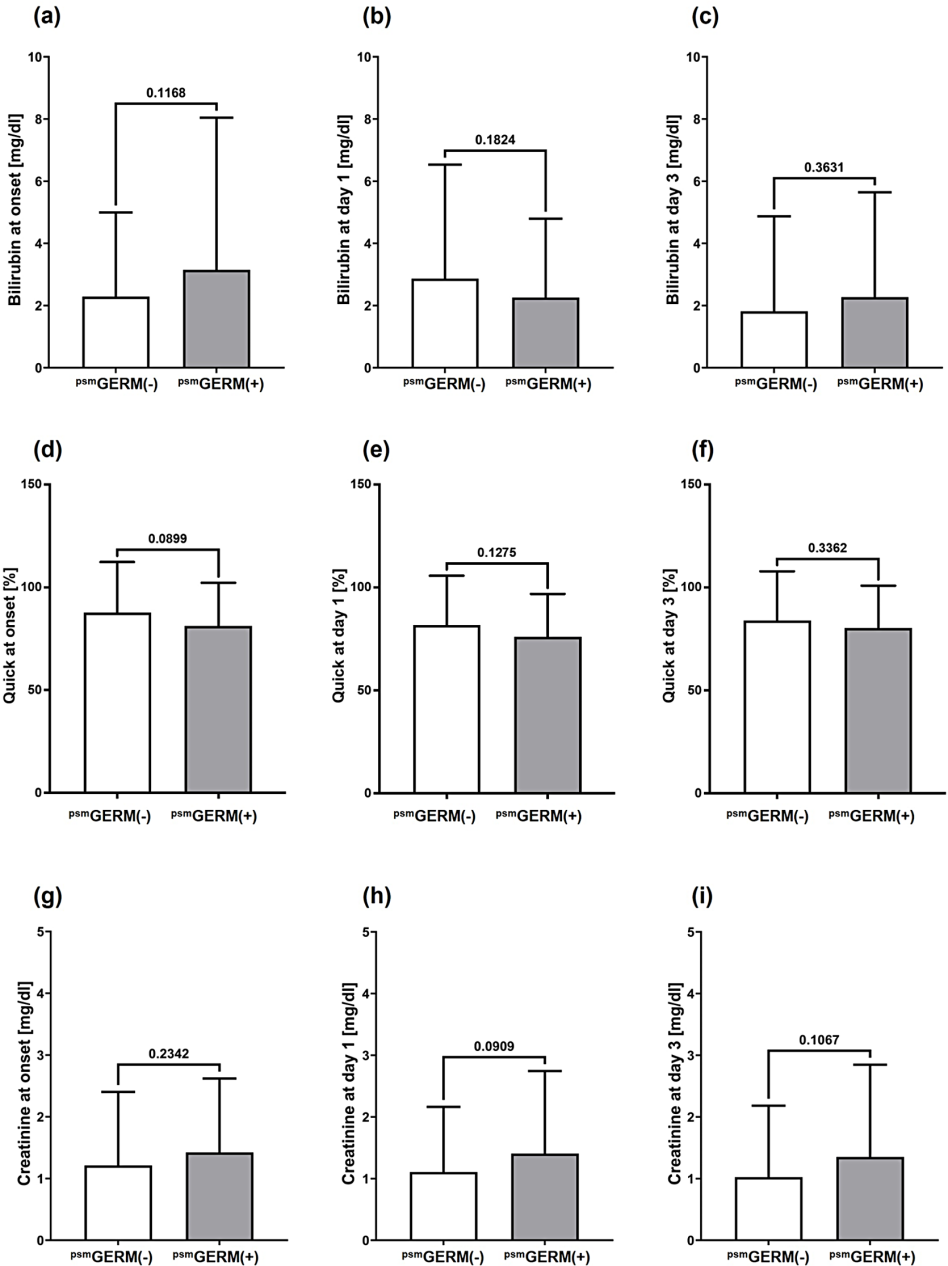
Serological markers for liver and kidney dysfunction. Columns indicate means and bars represent the respective standard deviations of serum total bilirubin **(a-c)**, prothrombin time in percentage, i.e. Quick **(d-f)** and serum creatinine **(g-i)** in peripheral blood of the propensity score matched (psm) patient cohorts without [^psm^GERM(-)] and with [^psm^GERM(+)] pathogen detection during acute pancreatitis therapy at onset of acute pancreatitis **(a**,** d**,** g)** and at in-hospital treatment day 1 **(b**,** e**,** h)** and day 3 **(c**,** f**,** i)**. The corresponding p values for each two-group comparison are indicated in the respective figures

To overcome initial imbalances between both groups, patients were matched using propensity score in a 1:2 ratio. Consequently, 123 patients from the GERM(-) group were matched with 74 patients from the GERM(+) group, resulting in balanced basic patient characteristics (Table 1). Especially severity and etiology of aP were equalized between the groups. However, even after propensity score matching, the serological markers of inflammation and infection, WBC and CRP, were markedly increased at onset and during the early treatment phase of aP (Fig. 1), whereas serological markers, specific for the hepato-biliary-pancreatic system, including total bilirubin, lipase and amylase, were not (Figs. 2 and 3).

Although in-hospital mortality did not differ between the two matched cohorts, patients from the ^psm^GERM(+) group had higher rates of fluid shifts with pleural effusions and ascites. They also had higher rates of intensive care treatment, longer stays in the ICU, and extended in-hospital stays (Table 1). Additionally, they exhibited a tendency toward lower Quick values as well as higher systemic creatinine values at onset of aP or during early treatment, respectively (Fig. 3), indicating organ failure and critical illness in these patients. Consequently, the composite outcome (length of in-hospital stay > 15 days or death), remained significantly different between the ^psm^GERM(-) and ^psm^GERM(+) group (31.7% versus 67.6%; Table 1).

**Fig. 4:**
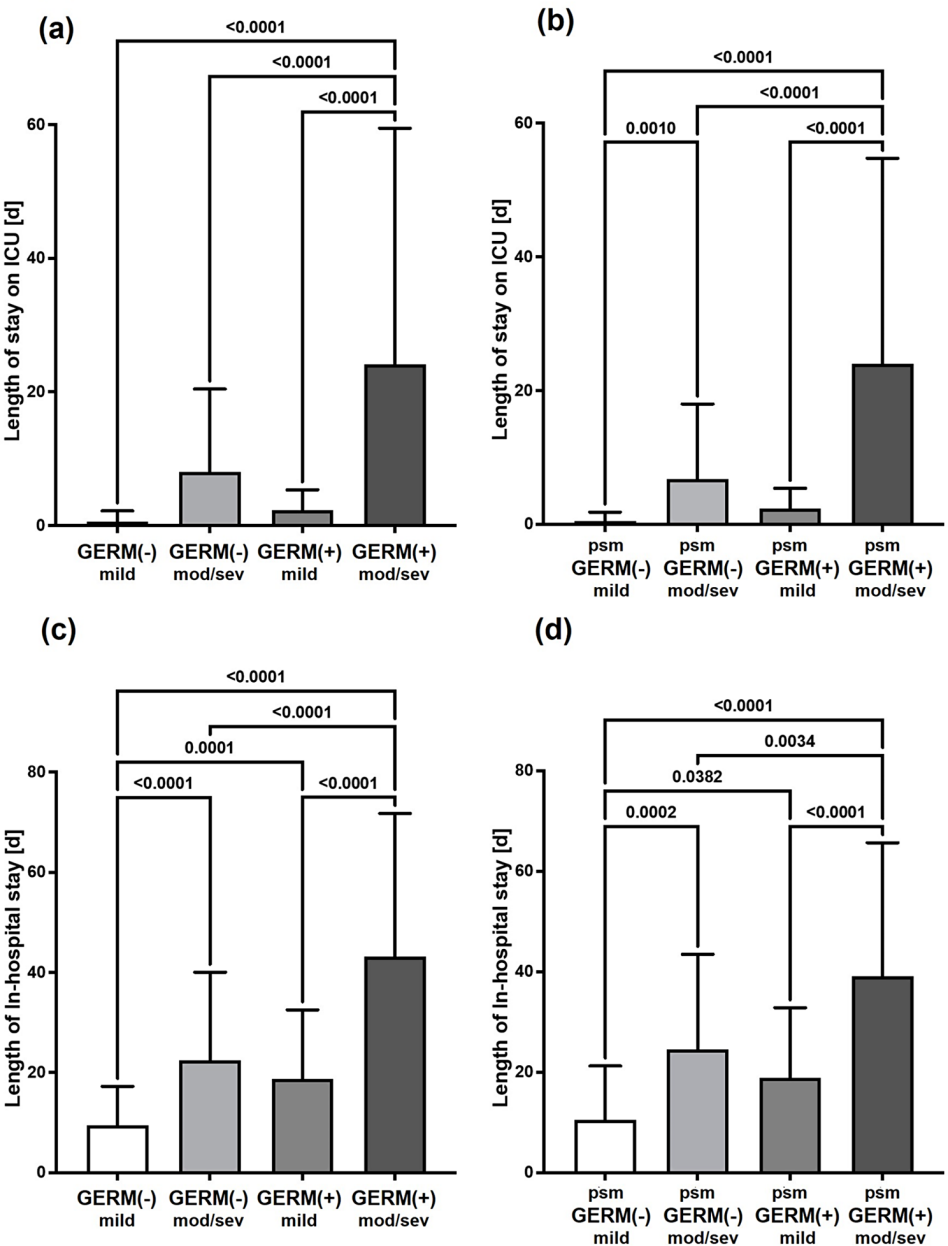
Lengths of hospital stay. Columns indicate means and bars represent the respective standard deviations. The total as well as propensity score matched (psm) patient cohorts without [GERM(-)] and with [GERM(+)] pathogen detection during acute pancreatitis therapy were subdivided regarding the severity of acute pancreatitis into patients with mild or moderate to severe (mod/sev) diseases concerning the revised Atlanta classification [2]. **a**,** c** depict the length of stay on intensive care unit (ICU) or total length of in-hospital stay of the total, unmatched patient cohorts. **b**,** d** depict the length of stay on intensive care unit (ICU) or total length of in-hospital stay of the propensity score matched patient cohorts. p values of multigroup comparisons < 0.05 are depicted

In the multi-group comparisons between patients with or without positive microbiology and stratified by clinical signs of either mild or moderate to severe aP according to the revised Atlanta classification [2], the stay in the ICU as well as the total duration of hospitalization were prolonged in GERM(+) patients with moderate to severe aP. Particularly, the duration in the ICU was significantly different compared to the GERM(+) counterparts with mild disease as well as to both GERM(-) patients with either mild or moderate to severe aP. These findings were consistent both before and after propensity score matching (Fig. 4).

### Early risk factors for bacterial load and invasive infection in acute pancreatitis

Two-group comparisons revealed a significant impact of bacterial load on outcomes of patients with aP. To further investigate early risk factors associated with microbial contamination and poorer outcomes, relevant demographic, etiological, and clinical variables from the early course of the disease were included into univariable and multivariable analyses. Therefore, rather than focusing on the severity or etiology of aP, elevated white blood cell counts at onset and the development of ascites during the early course of aP were evaluated as independent early predictors for microbial contamination, as shown in Table 2.

**Table 2:** Relevant predictors and risk factors associated with detection of pathogens

Variable	Univariable Analysis		Multivariable Analysis				
	*r* ^2^	*p* value	Coefficients			t	*p* value
			B	Std. Error	95% CI		
**Bacterial contamination**							
Sex	0.016	0.0782	-	-	-	-	-
Age [years]	0.010	0.1675	-	-	-	-	-
BMI [kg/m^2^]	0.001	0.7547	-	-	-	-	-
Moderate pancreatitis *	0.002	0.4903	-	-	-	-	-
Severe pancreatitis *	0.007	0.2553	-	-	-	-	-
Post-ERCP	0.002	0.5664	-	-	-	-	-
WBC [giga/l] onset	0.052	**0.0013**	0.015	0.006	0.004-0.027	2.636	**0.0091**
WBC [giga/l] day 1	0.035	0.0093	-	-	-	-	-
CRP [mg/l] onset	0.019	0.0521	-	-	-	-	-
CRP [mg/l] day 1	0.024	0.0315	-	-	-	-	-
Creatinine [mg/dl] onset	0.007	0.2342	-	-	-	-	-
Creatinine [mg/dl] day 1	0.015	0.0909	-	-	-	-	**-**
Lipase [U/l] onset	0.000	0.8943	-	-	-	-	-
Lipase [U/l] day 1	0.001	0.7141	-	-	-	-	-
Amylase [U/l] onset	0.000	0.8753	-	-	-	-	-
Amylase [U/l] day 1	0.000	0.8085	-	-	-	-	-
Biliary Etiology	0.002	0.4916	-	-	-	-	-
Pleural effusion	0.054	**0.0010**	0.086	0.077	-0.066-0.238	1.116	0.2657
Ascitic fluid	0.070	**0.0002**	0.198	0.077	0.047-0.349	2.589	**0.0104**
Volume therapy (first 24 h)	0.007	0.2511	-	-	-	-	-
Volume therapy (24–48 h)	0.005	0.3370	-	-	-	-	-
Volume therapy (after 48 h)	0.008	0.2370	-	-	-	-	-

Simple linear regression was used for univariable analysis and multiple linear regression for multivariable analysis to evaluate relevant early predictors and risk factors associated with bacterial load and consecutively worse clinical outcome. Variables with Spearman’s *r*² > 0.05 and *p* < 0.01 in univariable analysis were considered as relevant for inclusion into multivariable analysis. BMI = Body mass index; ERCP = Endoscopic retrograde cholangiopancreatography; WBC = White blood cell count; CRP = C-reactive protein

* In accordance with the revised Atlanta classification of 2012 [2]

### Pathogens from microbiological cultures of the acute pancreatitis patient cohort

**Fig. 5:**
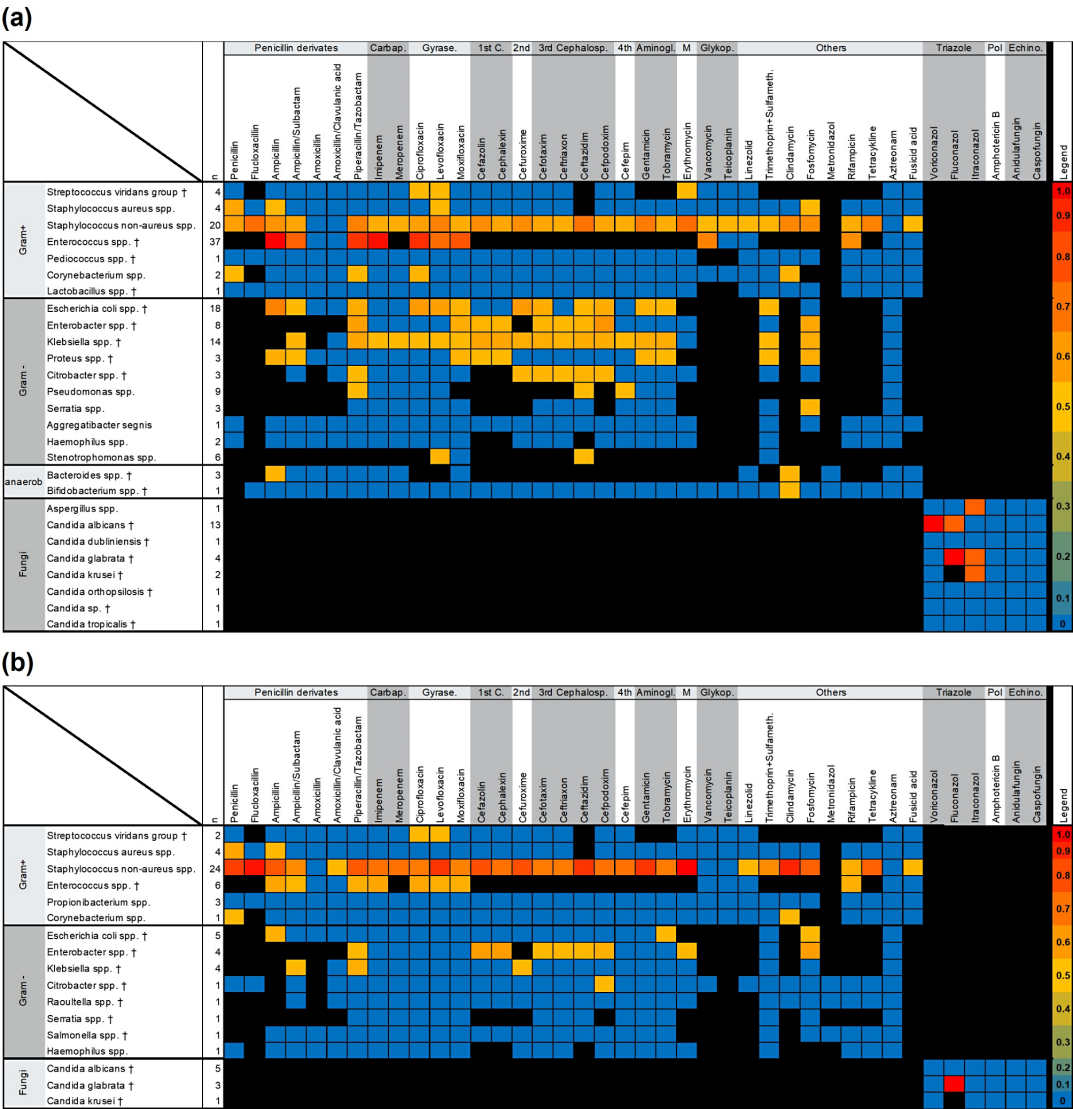
Heatmap of detected pathogens and corresponding acquired antibiotic or antifungal drug resistances. Colored boxes depict frequency distribution of acquired drug resistances of the detected pathogens from 0% in blue to 100% in red. Intrinsic resistances of the detected pathogens pursuant to the EUCAST (The European Committee on Antimicrobial Susceptibility Testing; http://www.eucast.org) documents are depicted as black boxes. **a** pathogens detected from local body fluids or (peri-) pancreatic necroses. **b** pathogens detected systemically from blood cultures. *Carbap* Carbapenem; *Gyrase* Gyrase inhibitor; *1st C.*,* 2nd*,* 3rd Cephalosp*,* 4th* 1st, 2nd, 3rd, 4th generation cephalosporin; *Aminogl.* Aminoglycosides; *M* Makrolide; *Glykop.* Glycopeptides; *Pol* Polyenes; *Echino.* Echinocandins; *Sulfameth.* Sulfamethoxazole; † Typical pathogens of the gut microbioma

The heatmap in Fig. 5 depicts all detected microorganisms from systemic blood cultures and local samples, including ascites, pleural effusion, (peri-) pancreatic necroses, and bile fluid, from the aP patient cohort. In both blood cultures and other samples, commensals of the physiological intestinal microbiome were the most frequent. Of the 67 organisms detected in blood cultures, 34 (50.7%) were attributed to gastrointestinal tract commensals. Similarly, 116 out of 164 (70.7%) organisms detected from other materials were of intestinal origin.

Enterococcus species and non-aureus Staphylococci were the most frequently identified pathogens, both demonstrating high rates of acquired drug resistance. Among gram-negative bacteria, Escherichia and Klebsiella species were predominant, with particularly high rates of drug resistance observed in E. coli species. Fungi were detected less frequently, with 13.4% at the systemic level in blood cultures and in 14.6% locally.

## Discussion

The present study highlights significantly poorer outcomes in patients with aP who develop invasive infections, as indicated by the detection of pathogens in either blood cultures or other body fluids and pancreatic necroses. Pathogen detection was associated with higher rates of critical illness, prolonged ICU stays and extended in-hospital stays.

It is known that infectious complications in aP, including infected pancreatic necroses and extrapancreatic infections such as pneumonia or bacteremia, contribute to organ failure and increased mortality [7, 28–31]. Similar findings, particularly in aP patients with extrapancreatic infections, were observed in the retrospective study by Jiang et al. [31]. This underscores the critical importance of initiating early, targeted anti-infective therapy in high-risk patients at the point of care. However, positive microbial identification in clinical practice can be challenging, particularly during the early phase of aP when clear clinical signs of infectious complications or sepsis may not yet be apparent. Early predictors for septic progression at the onset or during the initial phase of aP are largely lacking [13, 18]. Identifying high-risk patients for developing septic complications early in disease progression is crucial for prophylactic antibiotic treatment to prevent septic complications, potentially reducing morbidity, mortality, and prolonged ICU resource utilization and hospital stays [13]. Our multivariable analysis identifies two relevant predictors of bacterial load at the onset of aP: blood leukocytosis and the development of ascites. Both factors are easily assessable in clinical practice and provide strong indicators for initiating appropriate anti-infective therapy.

During the early phase of aP, mortality is often caused by sterile inflammation through SIRS, with organ failure driven by an overwhelming cytokine release [5]. Identifying patients at high risk for infection during the later course of severe aP is crucial, as CARS increases susceptibility to infections due to impaired intestinal barrier function and translocation of gut pathogens [5, 9, 32] into the bloodstream and/or locally into peritoneal fluid and necrotic (peri-) pancreatic tissue [28, 33, 34]. As a result, sepsis with organ failure leads to significant mortality [31, 35].

Prophylactic anti-infective strategies in aP are controversial [7, 13, 36–38]. While Ukai et al. and Ding et al. demonstrated lower rates of infected pancreatic necroses [38] and extrapancreatic infections [36], respectively, mortality was not improved in the more recent meta-analysis by Ding et al. [36]. In this context, Montravers et al. analyzed a multicentric cohort of patients admitted to ICU with aP and found that delayed initiation of anti-infective therapy was associated with higher rates of severe septic complications compared to early therapy. Their findings highlight the critical importance of timely intervention in managing infectious complications in aP, particularly in high-risk patient populations [39]. However, these studies did not evaluate early predictors of septic progression and did not stratify aP patients by early risk factors for infection, limiting the generalizability of their findings.

In the present study, we highlight ascitic fluid as an independent risk factor for bacterial load and invasive infection. In this line, ascites has previously been shown to be a negative prognostic factor in aP patients [40, 41]. The pathophysiology of ascites in aP is attributed to the severity of SIRS during the early phase and CARS in the late phase. Contributing factors include capillary leakage, fluid overload, hypoproteinemia, and local complications such as pancreatic duct injury, portal vein thrombosis, and obstructing peripancreatic fluid collections leading to lymphatic leakage [42–44]. Additionally, our analysis reveals that the translocation of pathogens, particularly those of gastrointestinal origin, into the abdominal cavity – due to impaired intestinal barrier function during the pathophysiologic course of aP [5, 9, 28, 32–34] – is associated with the development of ascites and poorer clinical outcomes. The second early predictor of bacterial load in our analysis was blood leukocytosis at the onset of aP, a well-known indicator of infectious courses, severe local and distant organ complications in aP patients [2, 4, 12, 45].

Our detailed data analysis shows that both predictors from the early onset of aP indicate an infectious complication in patients with aP. This suggests that prophylactic anti-infective therapy for these high-risk patients should be considered at an early stage of the disease, potentially reducing morbidity and mortality thereafter. Therefore, the detailed analysis of pathogens and their drug resistance profiles in the GERM(+) patient cohort, as shown in Fig. 5, holds significant clinical value. It supports the selection of appropriate antimicrobial therapy for high-risk patients and those with infectious complications of aP. The results of our pathogen and antibiotic susceptibility analysis align with findings from the large retrospective study by Wen et al. [46], which reported that early administration of broad-spectrum carbapenems in patients with severe biliary aP was associated with lower length of hospital stay and, most importantly, reduced mortality [46]. As our analysis indicates, a key reason for this could be the high susceptibility of the detected pathogens to carbapenems, which may help to prevent infectious and septic complications at the point of care by ensuring effective early antimicrobial coverage.

Despite its clinical relevance, our study has limitations. Overall, the retrospective, single-center study design limits the generalizability of the results. Furthermore, in our study, post-ERCP was the second most common cause of aP, surpassing biliary etiology. This may be attributed to more complex interventions performed in our tertiary center. The imbalanced patient cohort characteristics necessitating PSM further limit the unrestricted clinical applicability of our findings. Another limitation is the restricted availability of advanced laboratory parameters in this retrospective analysis from clinical routine data, such as procalcitonin [7]. Procalcitonin has been identified in the expert consensus on antibiotic therapy for acute pancreatitis as a potential biomarker to reduce unjustified antibiotic use [18]. Future prospective studies are needed to evaluate its predictive accuracy against our findings, specifically the predictive value of elevated WBC, for guiding early anti-infective decision-making. Nonetheless, our results warrant cautious consideration and verification in larger patient cohorts and form hypotheses for prospectively conduced trials to evaluate improvements in morbidity and mortality through prophylactic anti-infective therapy in high-risk patients, as defined by predictive factors from our multivariable analysis.

In conclusion, our findings underscore the hypothesis that pathogen detection in aP patients is associated with worsened clinical outcomes. Early predictive factors might enable timely initiation of prophylactic anti-infective therapy, potentially preventing septic complications and reducing morbidity and mortality in high-risk aP patients.

## Electronic supplementary material

Below is the link to the electronic supplementary material.

Supplementary Material 1: Supplement Fig. 1 Column bar graphs for markers for systemic inflammation or infection in the unmatched patient cohort without detection of pathogen [GERM(-)] and with [GERM(+)]. White blood cell count in peripheral blood [giga/l] at a onset, b day 1 and c day 3 and C-reactive protein [mg/l] at d onset, e day 1 and f day 3

Supplementary Material 2: Supplement Fig. 2 Column bar graphs for markers for pancreatic injury in the unmatched patient cohort without detection of pathogen [GERM(-)] and with [GERM(+)]. Lipase [U/l] at a onset, b day 1 and c day 3 and amylase [U/l] at d onset, e day 1 and f day 3

Supplementary Material 3: Supplement Fig. 3 Column bar graphs for markers for organ dysfunction in the unmatched patient cohort without detection of pathogen [GERM(-)] and with [GERM(+)]. Bilirubin [mg/dl] at a onset, b day 1 and c day 3, Quick’s-values [%] at d onset, e day 1 and f day 3 and creatinine [mg/dl] at g onset, h day 1 and i day 3

## Data Availability

Data available on reasonable request from the corresponding author.

## Abbreviations

aP: acute Pancreatitis
BMI: Body Mass Index
CARS: Compensatory Anti-inflammatory Response Syndrome
CRP: C-Reactive Protein
ERCP: Endoscopic Retrograde Cholangiopancreatography
EUCAST: The European Committee on Antimicrobial Susceptibility Testing
ICU: Intensive Care Unit
PSM: Propensity Score Matching
SIRS: Systemic Inflammatory Response Syndrome
WBC: White Blood Cell Count
