# Supplementary material for: Impact of invasive infections on clinical outcomes in acute pancreatitis: early predictive factors and implications for prophylactic anti-infective therapy
Source: Gut Pathog. 2025 Jan 19;17:5. doi: 10.1186/s13099-024-00671-3 (PMC11742995; doi:10.1186/s13099-024-00671-3)

F. Bender et al. Impact of Invasive Infections on Clinical Outcomes in Acute Pancreatitis: Early Predictive Factors and Implications for Prophylactic Anti-infective Therapy

For correspondence: Fabienne Bender, MD. fabienne.bender@chiru.med.uni-giessen.de

Supplement 2: Serum lipase and amylase as serological markers for acute pancreatitis. Columns indicate means and bars represent the respective standard deviations of serum lipase (a-c) and serum amylase values (d-f) in peripheral blood of the total, unmatched patient cohorts without [GERM(-)] and with [GERM(+)] pathogen detection during acute pancreatitis therapy at onset of acute pancreatitis (a, d) and at in-hospital treatment day 1 (b, e) and day 3 (c, f). The corresponding p values for each two-group comparison are indicated in the respective figures.

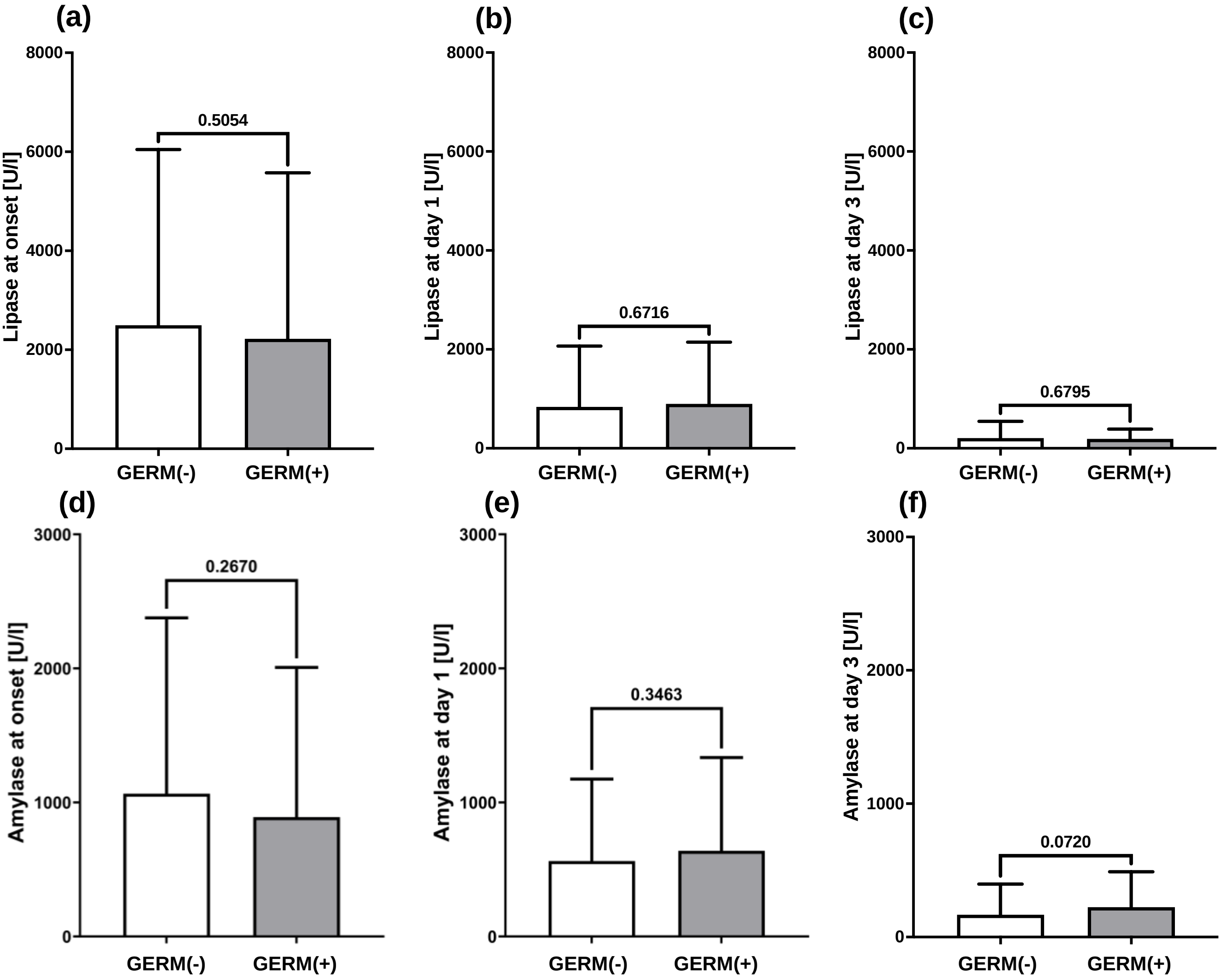

Supplement: Supplementary file 2 — Supplementary Material 2: Supplement Fig. 2 Column bar graphs for markers for pancreatic injury in the unmatched patient cohort without detection of pathogen [GERM(-)] and with [GERM(+)]. Lipase [U/l] at a onset, b day 1 and c day 3 and amylase [U/l] at d onset, e day 1 and f day 3 [file 13099_2024_671_MOESM2_ESM.pdf]
