# Supplementary material for: Impact of invasive infections on clinical outcomes in acute pancreatitis: early predictive factors and implications for prophylactic anti-infective therapy
Source: Gut Pathog. 2025 Jan 19;17:5. doi: 10.1186/s13099-024-00671-3 (PMC11742995; doi:10.1186/s13099-024-00671-3)

F. Bender et al. Impact of Invasive Infections on Clinical Outcomes in Acute Pancreatitis: Early Predictive Factors and Implications for Prophylactic Anti-infective Therapy

For correspondence: Fabienne Bender, MD. fabienne.bender@chiru.med.uni-giessen.de

Supplement 3: Serological markers for liver and kidney dysfunction. Columns indicate means and bars represent the respective standard deviations of serum total bilirubin (a-c), prothrombin time in percentage, i.e. Quick (d-f) and serum creatinine (g-i) in peripheral blood of the total, unmatched patient cohorts without [GERM(-)] and with [GERM(+)] pathogen detection during acute pancreatitis therapy at onset of acute pancreatitis (a, d, g) and at in-hospital treatment day 1 (b, e, h) and day 3 (c, f, i). The corresponding p values for each two-group comparison are indicated in the respective figures.

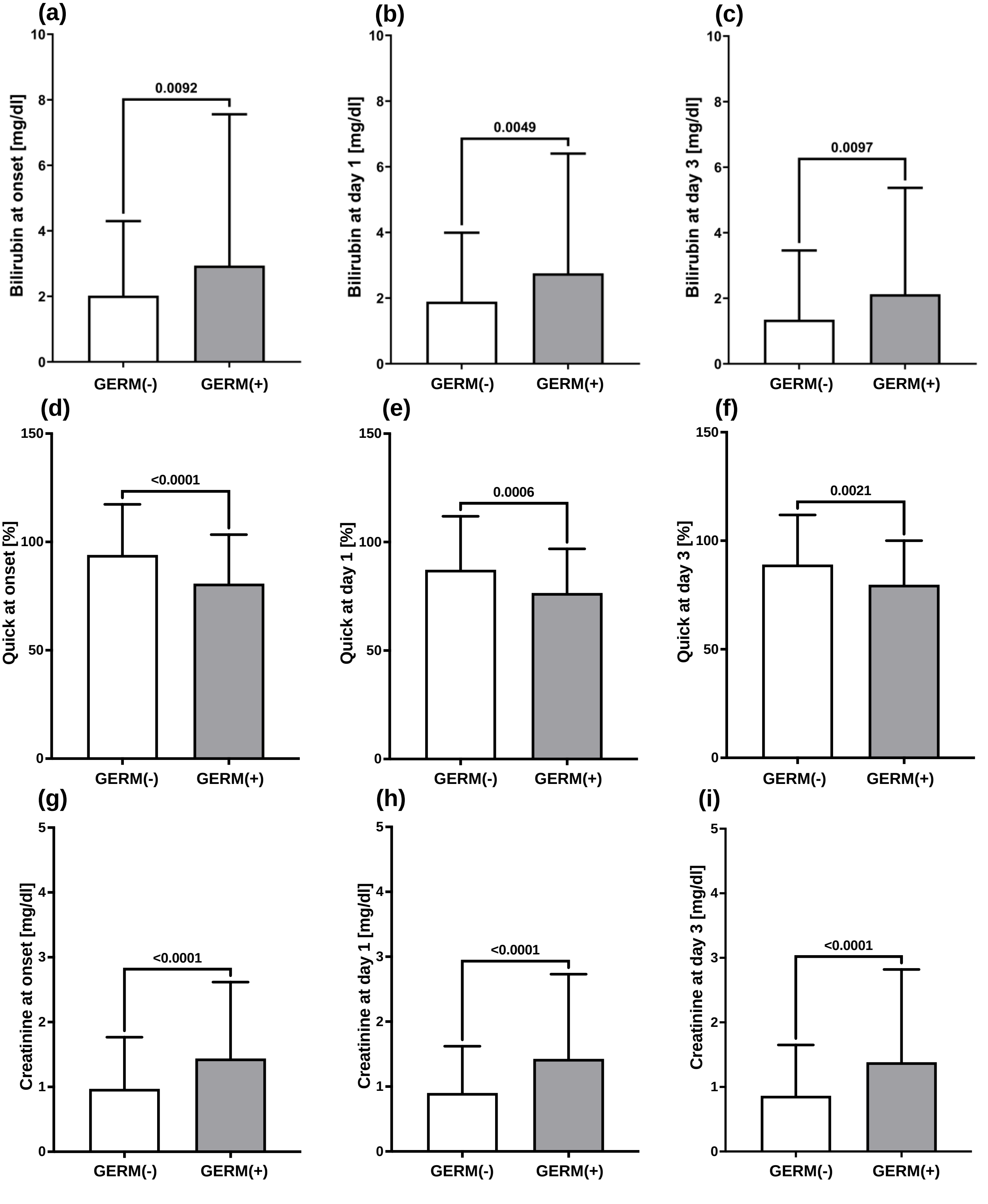

Supplement: Supplementary file 3 — Supplementary Material 3: Supplement Fig. 3 Column bar graphs for markers for organ dysfunction in the unmatched patient cohort without detection of pathogen [GERM(-)] and with [GERM(+)]. Bilirubin [mg/dl] at a onset, b day 1 and c day 3, Quick’s-values [%] at d onset, e day 1 and f day 3 and creatinine [mg/dl] at g onset, h day 1 and i day 3 [file 13099_2024_671_MOESM3_ESM.pdf]
